# Supplementary material for: An Integrated Systems Biology Approach Identifies the Proteasome as A Critical Host Machinery for ZIKV and DENV Replication
Source: Genomics Proteomics Bioinformatics. 2021 Feb 19;19(1):108–22. doi: 10.1016/j.gpb.2020.06.016 (PMC8498969; doi:10.1016/j.gpb.2020.06.016)
Supplement: Supplementary Figure S1 — Preparation of fluorescent-labeled ZIKV andDENV proteins A. Flow chart of fluorescent-labeled protein probes. B. Examples of successful PCR amplifications of ORFs using ZIKV cDNA templates. C. Examples of entry clones digested by BsrGI to release correct-size ORFs and then detected by 1% agarose gel. D. Examples of destination clones digested by BsrGI to release correct-size ORFs and then detected by 1% agarose gel. E. Examples of successful Cy5-labeled ZIKV and DENV protein probes detected by SDS-PAGE gel. Blue arrows indicate each protein’s probe with correct molecular weight. [file mmc1.pptx]

## Slide 1
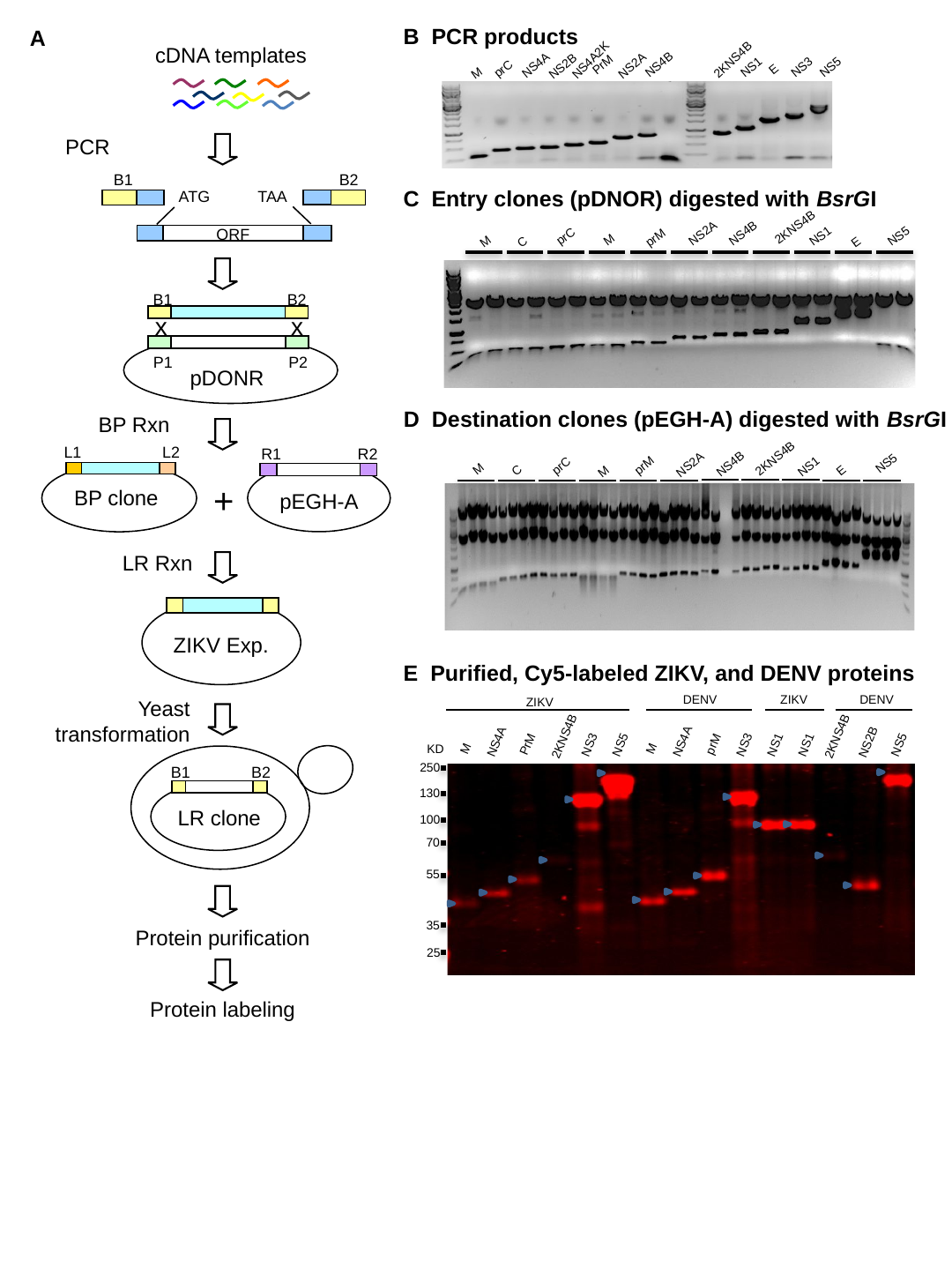

B PCR products
A
cDNA templates
2KNS4B
NS4A2K
PrM
NS4B
NS4A
NS2A
NS2B
NS1
NS3
NS5
prC
E
M
PCR
B2
B1
TAA
ATG
ORF
C Entry clones (pDNOR) digested with BsrGI
2KNS4B
NS2A
NS4B
NS1
NS5
prC
prM
M
M
C
E
B1
B2
x
x
P1
P2
pDONR
D Destination clones (pEGH-A) digested with BsrGI
BP Rxn
L1
L2
R2
R1
2KNS4B
NS5
NS4B
NS2A
prM
prC
NS1
M
C
E
M
+
BP clone
pEGH-A
LR Rxn
ZIKV Exp.
E Purified, Cy5-labeled ZIKV, and DENV proteins
DENV
ZIKV
DENV
ZIKV
2KNS4B
2KNS4B
NS4A
NS4A
NS2B
prM
PrM
NS3
NS5
NS3
NS1
NS1
NS5
M
M
KD
250
130
100
70
55
35
25
Yeast transformation
B1
B2
LR clone
Protein purification
Protein labeling
