# Supplementary material for: An Integrated Systems Biology Approach Identifies the Proteasome as A Critical Host Machinery for ZIKV and DENV Replication
Source: Genomics Proteomics Bioinformatics. 2021 Feb 19;19(1):108–22. doi: 10.1016/j.gpb.2020.06.016 (PMC8498969; doi:10.1016/j.gpb.2020.06.016)
Supplement: Supplementary Figure S2 — ZIKV– and DENV–host PPIs A. Interaction specificity of host proteins with viral proteins. Approximately 40% of human proteins identified by the PPI analysis only interacted with one or two viral proteins, while 24 human proteins interacted with all of the viral proteins tested and were removed from further analysis. B. Comparison of human proteins identified in this study with those known to be targeted by viruses in the VirusMINT and Virhostome databases. C. Comparison of human proteins identified in this study with a recently published data associated ZIKV-Human PPI based on MS method, which identified 701 and 688 human binding proteins by IP-MS and BioID, respectively. D. The host proteins that interacted with both ZIKV and DENV proteins are shown in the middle of the PPI network, and the host proteins that interact specifically to either ZIKV or DENV proteins are placed on top or bottom of the network. [file mmc2.pptx]

## Slide 1
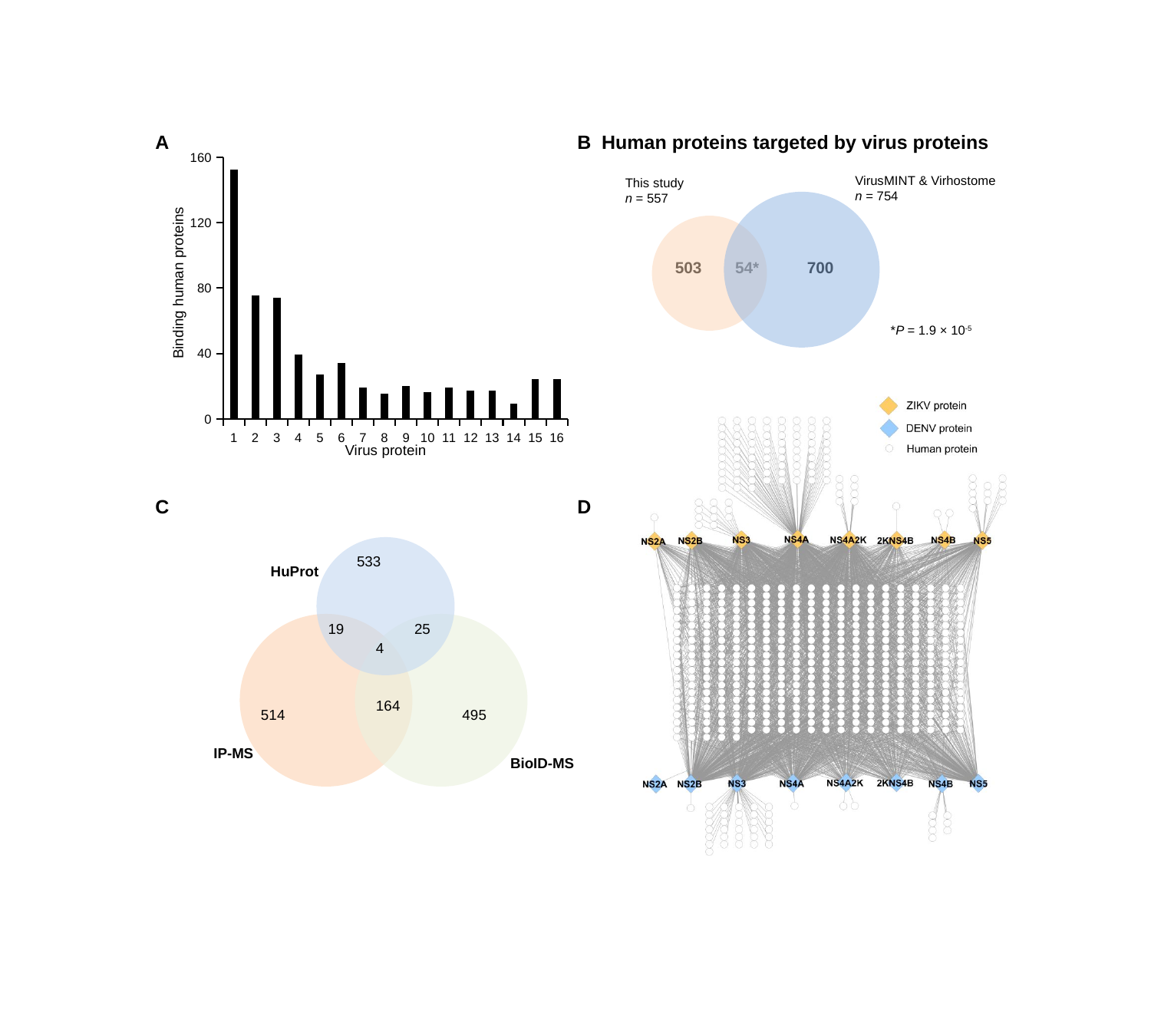

A
B Human proteins targeted by virus proteins
VirusMINT & Virhostome
n = 754
This study
n = 557
503
54*
700
*P = 1.9 × 10^-5^
### Chart
| Category | |
|---|---|
| 1 | 152.0 |
| 2 | 75.0 |
| 3 | 74.0 |
| 4 | 39.0 |
| 5 | 27.0 |
| 6 | 34.0 |
| 7 | 19.0 |
| 8 | 15.0 |
| 9 | 20.0 |
| 10 | 16.0 |
| 11 | 19.0 |
| 12 | 17.0 |
| 13 | 17.0 |
| 14 | 9.0 |
| 15 | 24.0 |
| 16 | 24.0 |Binding human proteins
Virus protein
C
D
533
HuProt
19
25
4
164
514
495
IP-MS
BioID-MS
