# Supplementary material for: An Integrated Systems Biology Approach Identifies the Proteasome as A Critical Host Machinery for ZIKV and DENV Replication
Source: Genomics Proteomics Bioinformatics. 2021 Feb 19;19(1):108–22. doi: 10.1016/j.gpb.2020.06.016 (PMC8498969; doi:10.1016/j.gpb.2020.06.016)

NS-1 protein level in HEK293 cells

Compound cytotoxicity in HEK293 cells

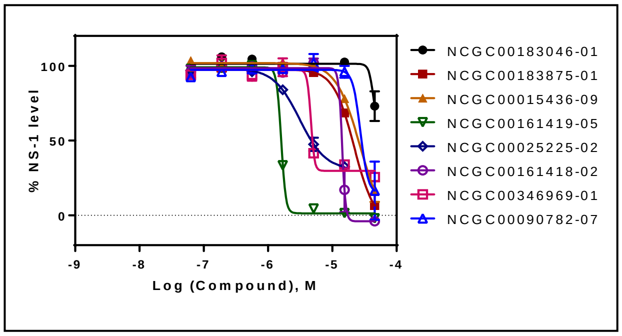

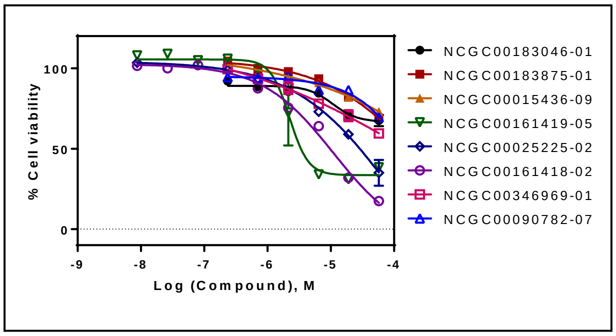

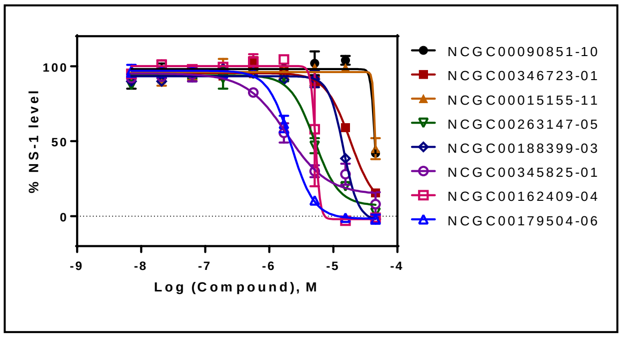

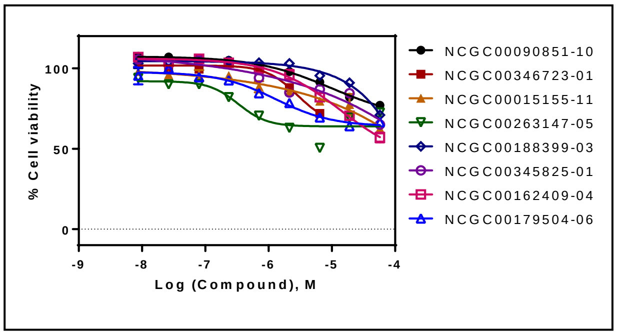

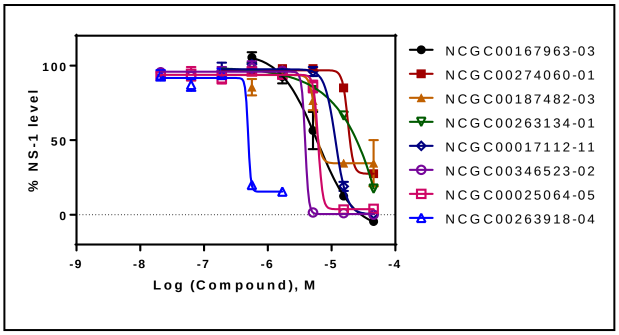

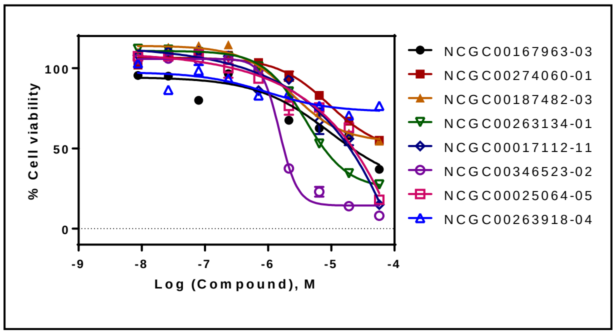

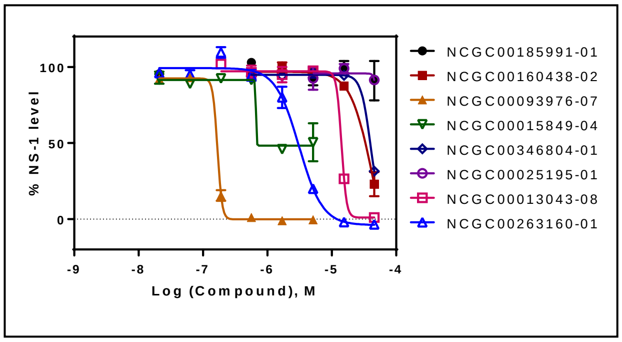

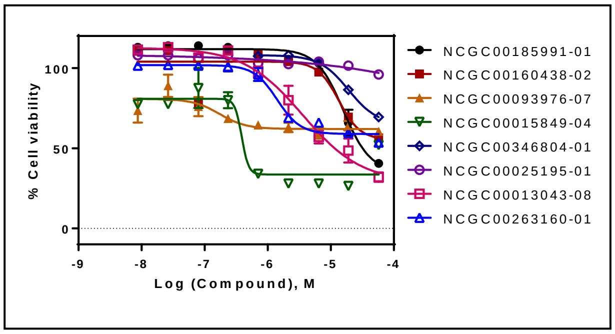

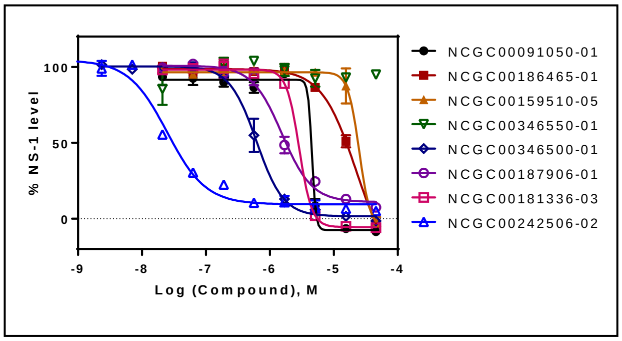

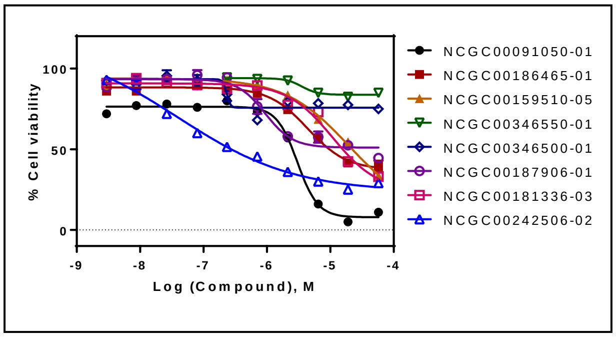

NS-1 protein level in HEK293 cells

Compound cytotoxicity in HEK293 cells

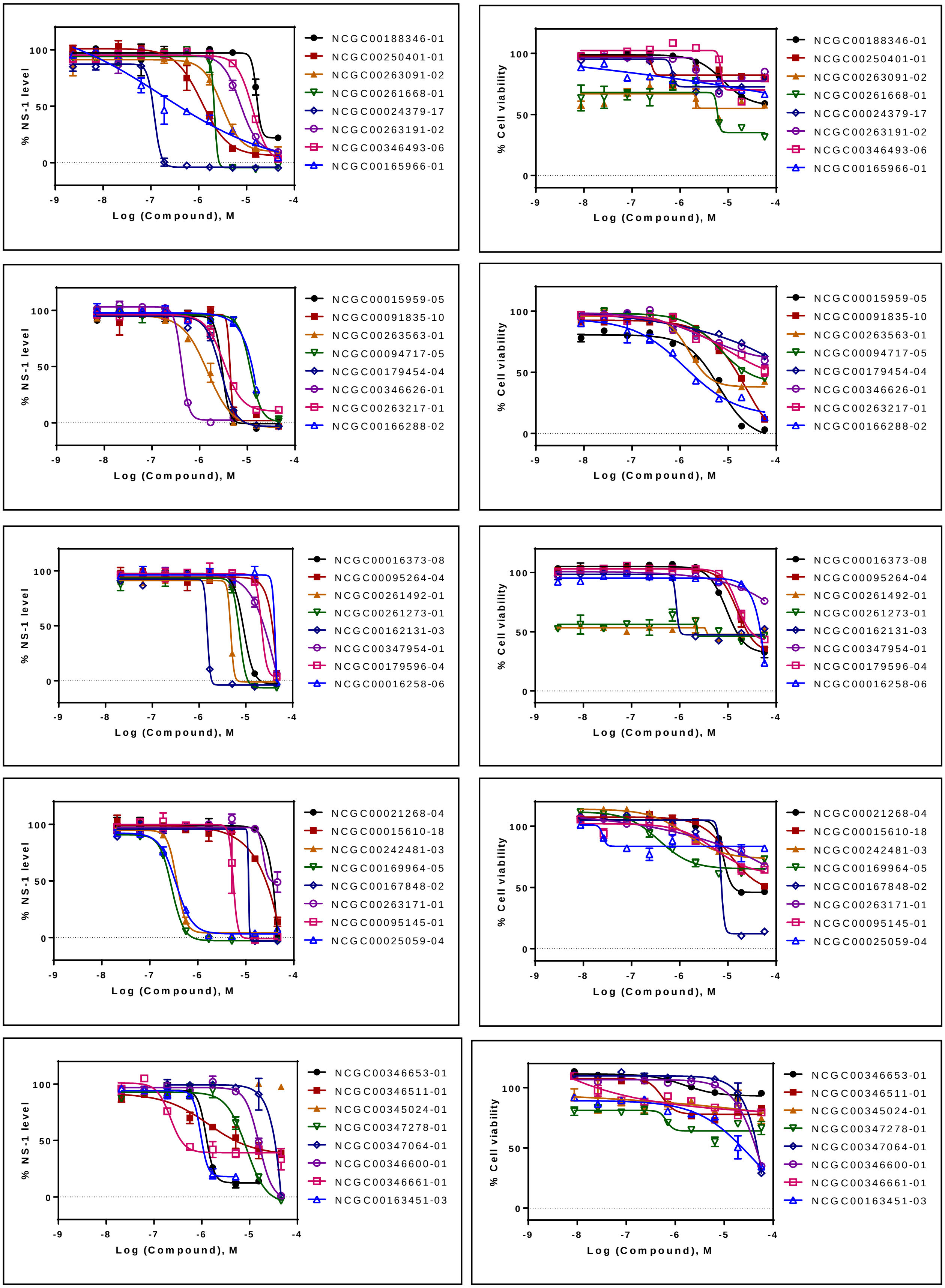

NS-1 protein level in HEK293 cells | Compound cytotoxicity in HEK293 cells

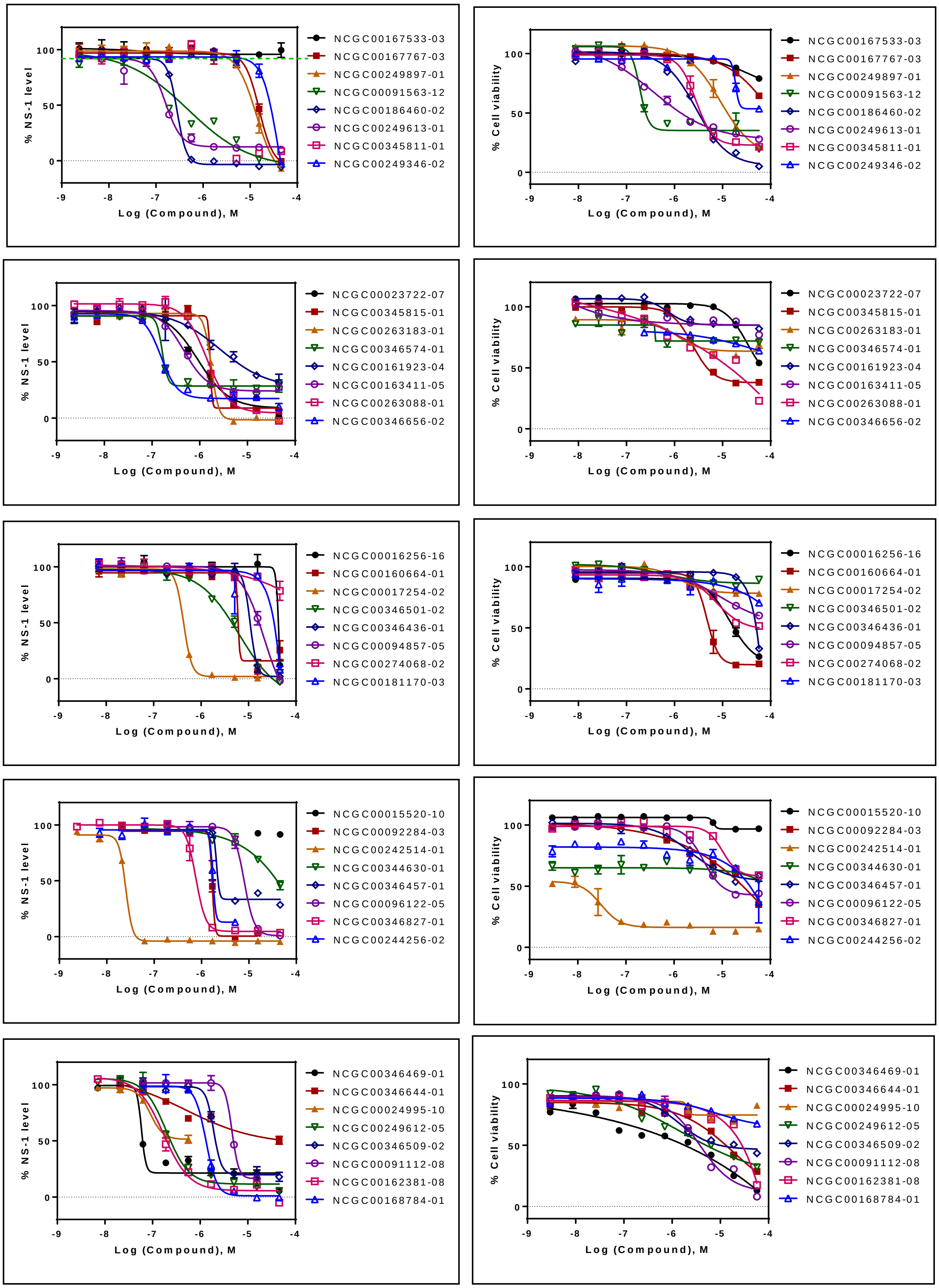

NS-1 protein level in HEK293 cells

Compound cytotoxicity in HEK293 cells

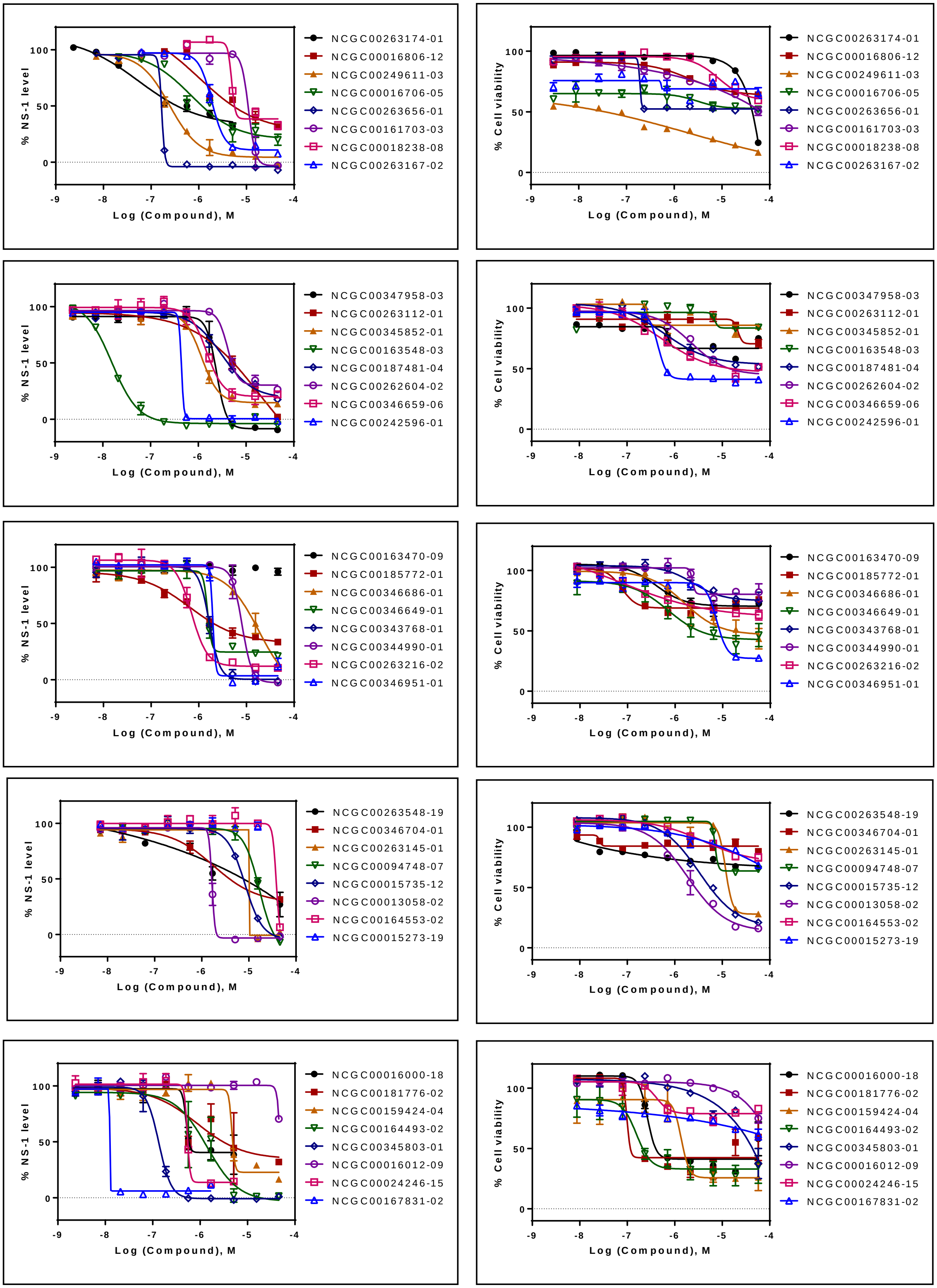

NS-1 protein level in HEK293 cells

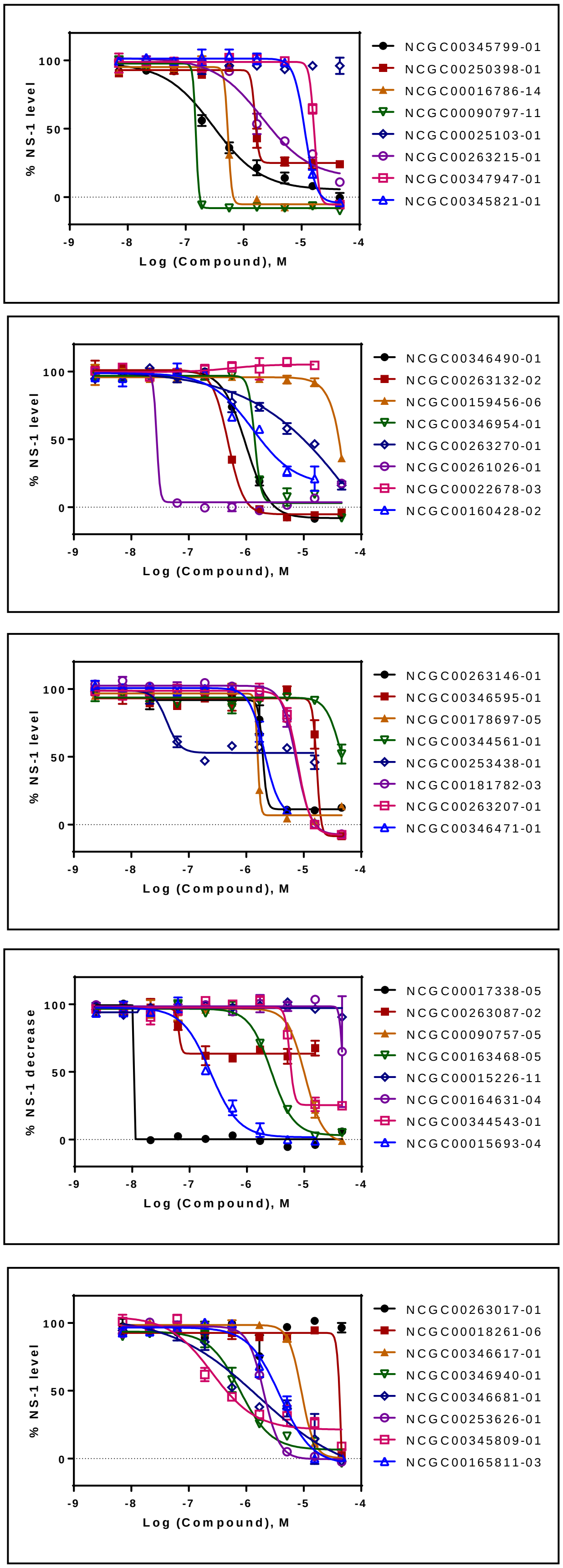

Compound cytotoxicity in HEK293 cells

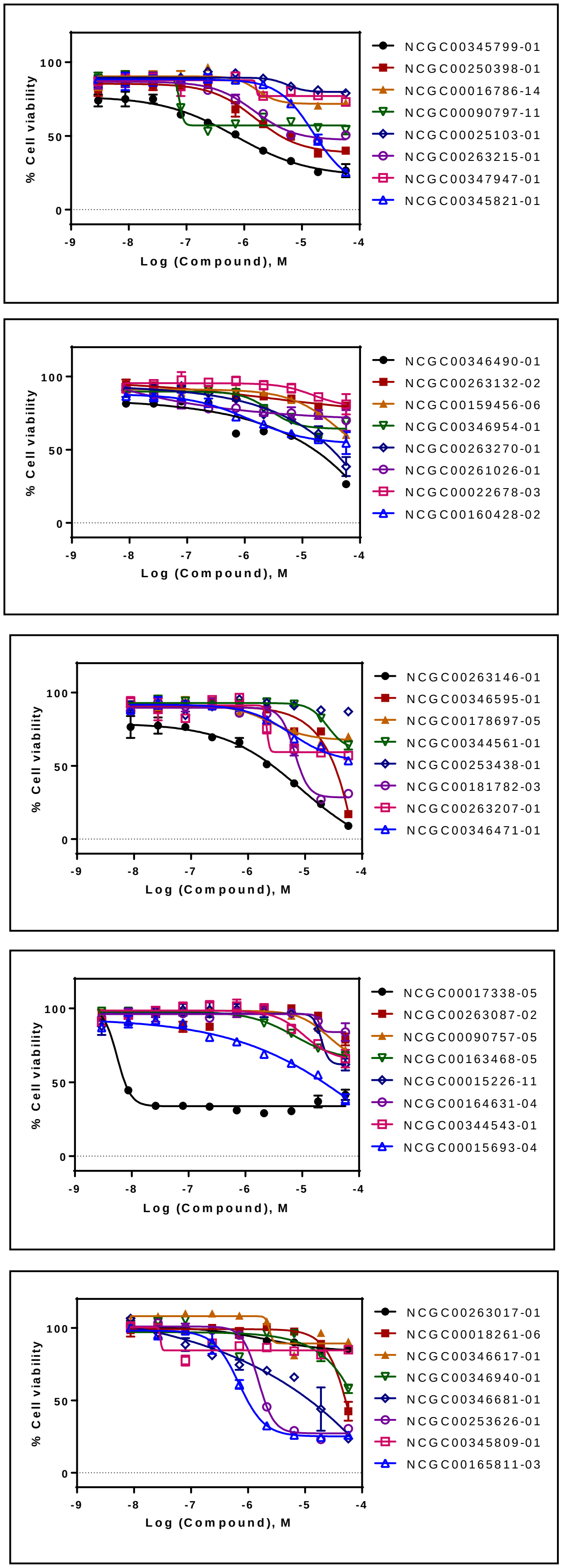

NS-1 protein level in HEK293 cells

Compound cytotoxicity in HEK293 cells

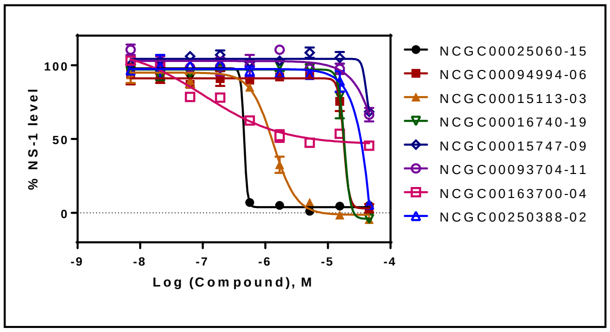

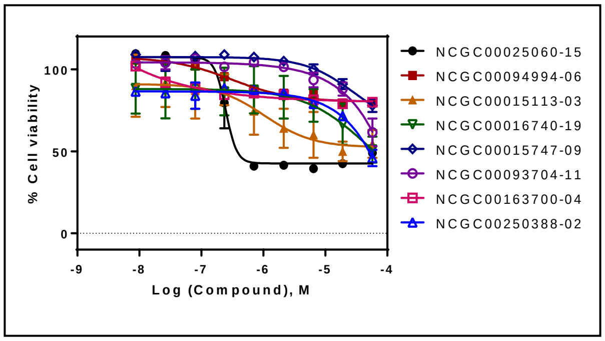

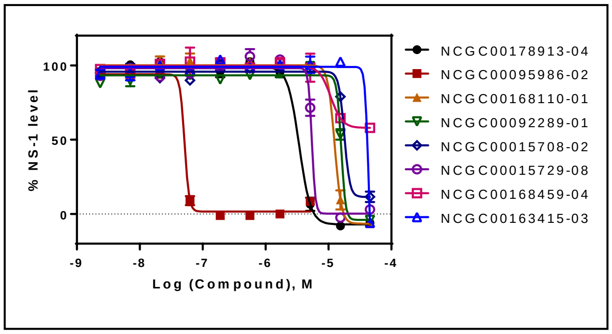

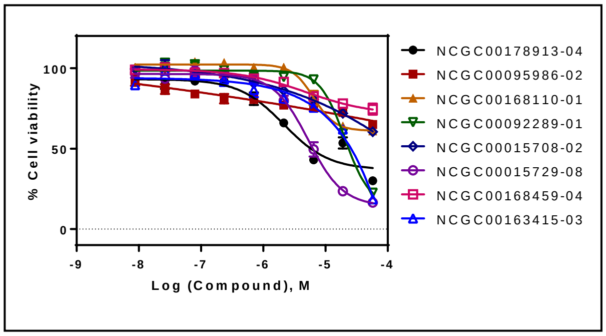

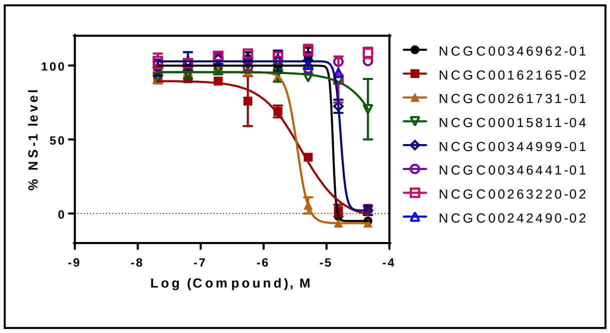

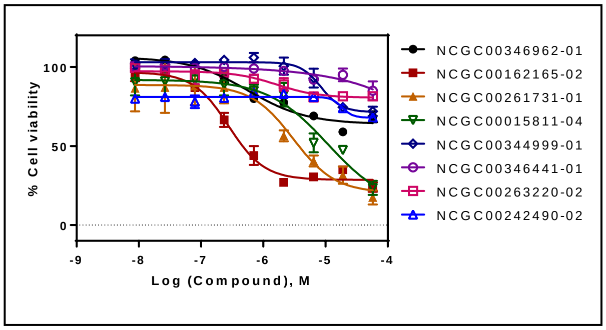

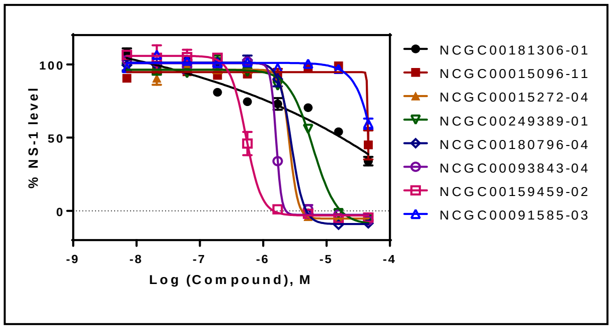

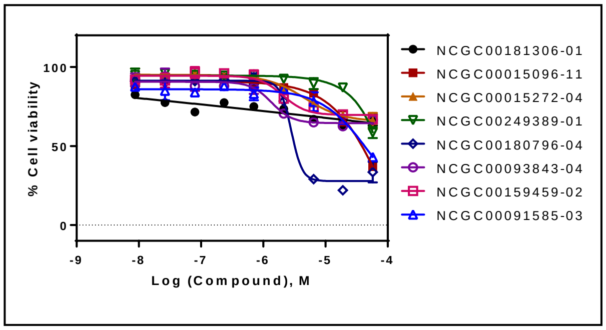

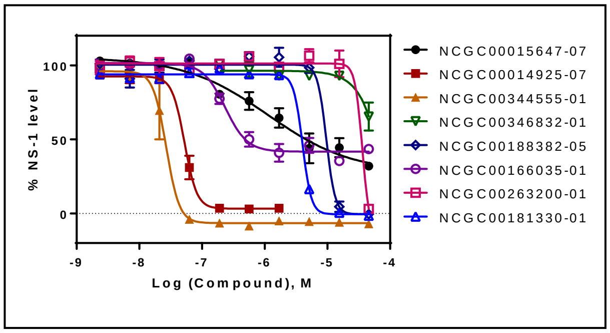

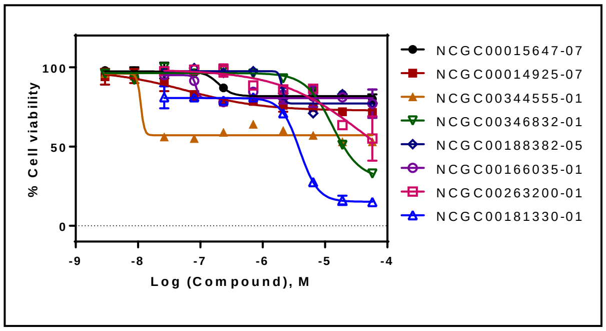

NS-1 protein level in HEK293 cells

Compound cytotoxicity in HEK293 cells

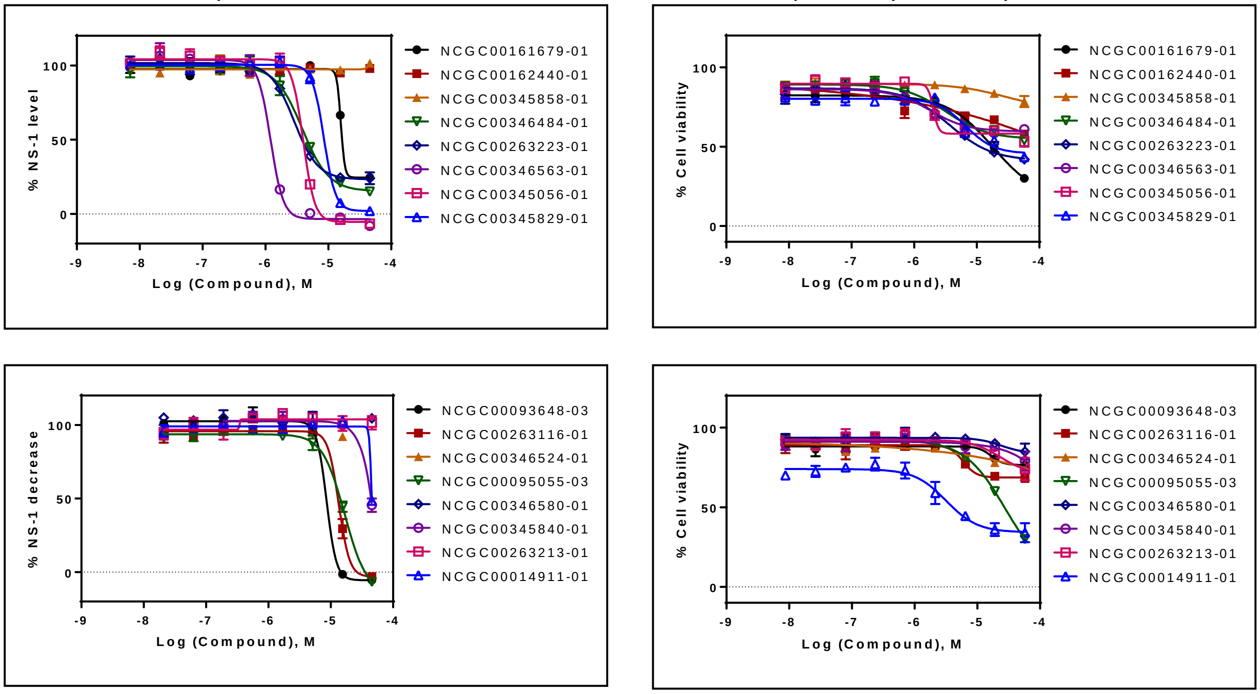

Supplement: Supplementary Figure S3 — Inhibitory activityand cytotoxicity analysisof256 preliminary hitsagainst ZIKV [file mmc3.pdf]
