# Supplementary figures and images for: An Integrated Systems Biology Approach Identifies the Proteasome as A Critical Host Machinery for ZIKV and DENV Replication
Source: Genomics Proteomics Bioinformatics. 2021 Feb 19;19(1):108–22. doi: 10.1016/j.gpb.2020.06.016 (PMC8498969; doi:10.1016/j.gpb.2020.06.016)

**A**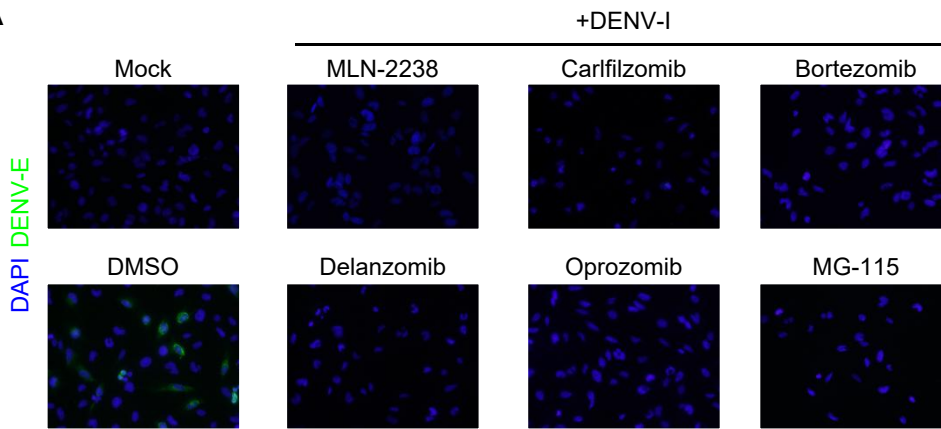**B**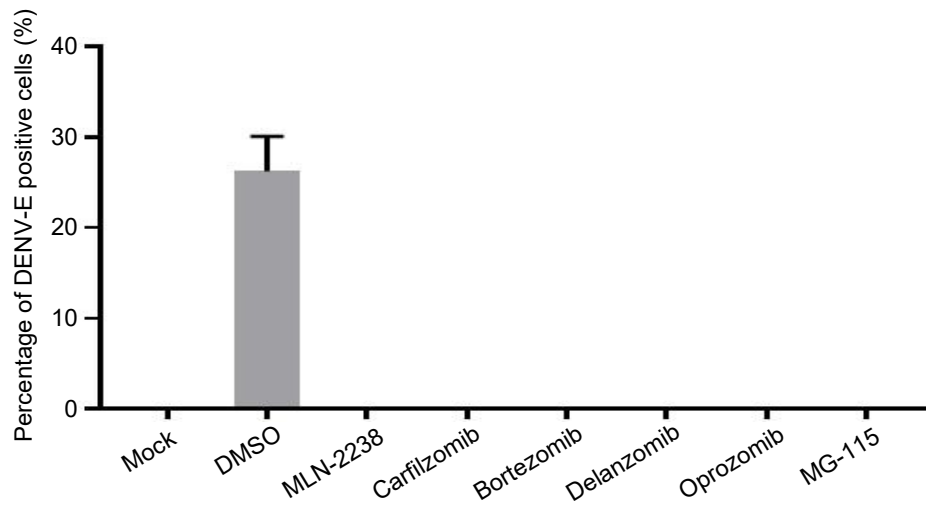

Supplement: Supplementary Figure S4 — Proteasome inhibitors inhibit the DENV production A. The SNB-19 cells were infected by DENV (MOI = 1) in the presence of 1 µM of each inhibitor and then incubated for 48 h for immunocytochemistry of DENV envelope (DENV-E) protein. B. Quantification of inhibition of DENV production by proteasome inhibitors as in (A). Values represent mean + SD (n = 3). [file mmc4.pdf]
